# Punicalagin Reversed the Hepatic Injury of Tetrachloromethane by Antioxidation and Enhancement of Autophagy

**DOI:** 10.1089/jmf.2019.4411

**Published:** 2019-12-11

**Authors:** Jingfang Luo, Yi Long, Guofeng Ren, Yahui Zhang, Jihua Chen, Ruixue Huang, Lina Yang

**Affiliations:** ^1^ Department of Nutrition and Food Hygiene, Xiangya School of Public Health, Central South University, Changsha, Hunan, China.; ^2^ Children's Medical Center, Hunan Provincial People's Hospital, Changsha, Hunan, China.; ^3^ Department of Occupational and Environmental Health, Xiangya School of Public Health, Central South University, Changsha, Hunan, China.

**Keywords:** antioxidation, autophagy, liver injury, polyphenols, pomegranate, punicalagin

## Abstract

Hepatic injury is significant in the pathogenesis and development of many types of liver diseases. Punicalagin (PU) is a bioactive antioxidant polyphenol found in pomegranates. To explore its protective effect against carbon tetrachloride (CCl_4_)-induced liver injury and the mechanism, Institute of Cancer Research (ICR) mice and L02 cells were used to observe the changes of serum biochemical indicators, histopathological liver structure, cell viability, antioxidative indices, and autophagy-related proteins were assessed. In ICR mice, PU ameliorated the CCl_4_-induced increase of the serum aspartate aminotransferase, alanine aminotransferase, the activity of liver lactate dehydrogenase, and the damage of histopathological structure, and exhibited a hepatoprotective effect against CCl_4_. PU attenuated oxidative stress by decreasing the liver malondialdehyde level and increasing the activities of liver superoxide dismutase, glutathione peroxidase, and the expression of the liver nuclear factor E2-related factor (Nrf2) protein. Furthermore, according to the vivo and vitro experiments, PU might activate autophagy through the mediation of the Akt/FOXO3a and P62/Nrf2 signaling pathway. Taken together, these results suggest that PU may protect against CCl_4_-induced liver injury through the upregulation of antioxidative activities and autophagy.

## Introduction

Liver is an important organ for metabolism of drugs and chemicals, while liver disease has been a major global health problem. Some extrahepatic factors, such as alcohol, toxins, and drugs, can lead to liver injury, and liver injury is one of the pathological bases of liver cirrhosis or liver failure.^1^ Therefore, the prevention of liver injury is essential. Recently, searching for phytochemicals extracted from natural food, which can prevent and control liver injury and liver diseases with high efficacy and low toxicity, is of significant interest.

In this study, we focus primarily on the effect of punicalagin (PU), one of the major polyphenols isolated from pomegranates, on liver injury. It has antioxidant, anti-inflammatory, anticancer, hepatoprotective, and antigenotoxic activities.^2^ Its health benefits, especially those attributed to antioxidation, have been well demonstrated. Fouad *et al.*^3^ reported that PU alleviated the cyclophosphamide-induced liver injury in rats due to its antioxidative activity, followed by reduced levels of serum ALT, nuclear factor kappa-light-chain-enhancer of activated B cells (NF-*κ*B), tumor necrosis factor-*α* (TNF-*α*), and interleukin 1 (IL-1).^3^ PU also exhibited antioxidant activities in murine macrophages, inducing IL-10 secretion and the phosphorylation of Akt and STAT.^4^ Lin *et al.* found that PU exhibited antioxidant activities in an acetaminophen-induced model of liver injury.^5^ Lin *et al.* have reported that PU had antioxidant and hepatoprotective activities in rats with carbon tetrachloride (CCl_4_)-induced liver damage.^6^

In addition to antioxidation, autophagy PU was reported to protect against physiological and pathological abnormalities of liver. It could relieve liver from the injury caused by alcohol and Cytochrome P450 (CYP450)-dependent oxidative stress.^7^ Autophagy is divided into three types: macroautophagy, microautophagy, and chaperone-mediated autophagy. Macroautophagy is characterized by the double-membrane autophagosomes, cleaning the destroyed or aged organelle and proteins.^8^ Shi *et al.* found that augmenter of liver regeneration could ameliorate CCl_4_-induced liver injury through promoting autophagy in mice.^9^

Some evidence also proved that PU could regulate autophagy against oxidative stress or apoptosis in some other diseases.^10–12^ The combined evidence led to the hypothesis that PU may protect hepatocytes from CCl_4_ toxicity through the autophagy pathway. The related mechanism has not been clearly defined, and no study explored the related mechanism of this protective effect of PU involving the P62/nuclear factor E2-related factor (Nrf2)/Kelch-like ECH-associated protein 1 (Keap1) pathway, autophagy, and the Akt/forkhead box O 3a (FOXO3a) pathway.

In this study, we explored this potential mechanism in an animal model of CCl_4_-induced liver injury. CCl_4_-induced cell injury was evaluated in L02 cells, and it was the first time to examine if PU can protect hepatocytes by regulating the p62/Nrf2/Keap1 pathway, autophagy, and the Akt/FOXO3a pathway. It is likely to provide new clues to identify novel approaches to effectively protect against liver injury.

## Materials and Methods

### Reagents

PU (CAS: 65995-63-3, purity = 78% for animal treatment and 98% for cell treatment) was acquired from Chengdu Herbpurify Co. Ltd. (Chengdu, China). CCl_4_ was purchased from Tianjin Guangfu Technology Development Co. Ltd. (Shanghai, China). Vegetable oil purchased from Hunan Jinhao Camellia Oil Co. Ltd. (Hunan, China). Malondialdehyde (MDA), superoxide dismutase (SOD), glutathione peroxidase (GSH-Px), and lactate dehydrogenase (LDH) detection kits were provided by the Nanjing Jiancheng Bioengineering Institute (Nanjing, China). Proteins were extracted using a tissue protein extraction kit (Beyotime Biotechnology, Beijing, China). MTT assay kits were purchased from Ding Guo Changsheng Biotechnology Co. Ltd. (Beijing, China).

Antibodies against P62 (#18420-1-AP), Beclin1 (#11306-1-AP), LC3B (#18725-1-AP), Nrf2 (#16396-1-AP), Akt phosphorylation (p-Akt) (#66444-1-Ig), and *β*-actin (mouse polyclonal) were purchased from Proteintech, Inc. (Wuhan, China). Antibodies against phosphorylation of FOXO3a (p-FOXO3a) were purchased from Cell Signaling Technology, Inc. (Danvers, MA, USA). Secondary antibodies were purchased from ZSGB-BIO Biotechnology Co. Ltd. (Beijing, China). Membrane blots were developed using the BeyoECL Star system (Beyotime Biotechnology).

### Animals and experimental design

Fifty healthy male Institute of Cancer Research (ICR) mice (20 ± 2 g) at 4–5 weeks of age were provided by Changsha Tianqin Biotechnology Co. Ltd. (Changsha, China) (animal production license no. SCXK [to Xiang] 2014-0011). The mice were housed at a temperature of 22°C ± 2°C and a humidity of 50% ± 10% under a 12 h/12 h dark/light cycle, and had free access to food and water. All animal experiment procedures conformed to the National Institutes of Health Guide for the Care and Use of Laboratory Animals and were approved by the Xiangya School of Public Health Institutional Review Board, Central South University (No. XYGW-2016-05).

After 1 week of acclimatization, the mice were randomly divided into five groups, control group: mice were given daily double-distilled water by gavages; CCl_4_ group: mice were given daily double-distilled water by gavages; CCl_4_+PU1 group: mice were treated daily with PU (20 mg/kg) dissolved in distilled water; CCl_4_+PU2 group: mice were treated daily with PU (40 mg/kg) dissolved in distilled water; and CCl_4_+PU3 group: mice were treated daily with PU (80 mg/kg) dissolved in distilled water. All gavages were continued for 4 weeks with the dose of 0.1 mL/100 g body weight, and 1 h after the last gavage, all mice except those in the control group were intraperitoneally injected with 0.07% (v/v) CCl_4_ (0.1 mL/100 g body weight). After 16 h, the blood samples and liver tissues were collected. Blood was collected into sodium heparin tubes and stored at −20°C, whereas liver tissues were stored at −80°C.

### L02 cell culture and treatment

L02 cells were obtained from Zhuohua Zhang research group of the Center for Medical Genetics, School of Life Sciences, Central South University, Changsha, Hunan, China. The cells were grown in RPMI 1640 with 10% fetal bovine serum (FBS) and a 1% penicillin/streptomycin solution (100 U/mL penicillin and 100 *μ*g/mL streptomycin) under 5% CO_2_ humidified air at 37°C. Cells were plated in six-well plates with 4 × 10^5^ cells/well and incubated overnight to attain confluency. Then, the cells were treated with medium with or without 10 *μ*M chloroquine (CQ) for 2 h, followed by 50 *μ*M PU in the PU group and 10 *μ*M CQ group incubated for 24 h. After that, the cells in CCl_4_ group, PU group, and CQ group were treated with 30 *μ*M CCl_4_ (dissolved in 0.1% DMSO in 1640 without FBS), and the control group was treated with 0.1% DMSO for another 2 h.

### Serum assays

Serum aspartate aminotransferase (AST) and alanine aminotransferase (ALT) levels were measured using an Olympus Chemistry Analyzer Model AU2700 (Olympus Optical Co. Ltd., Tokyo, Japan). The levels of MDA and the activities of SOD, GSH-Px, and LDH in liver were measured using kits according to the instructions of the manufacturers.

### Hepatic histopathological evaluation and examination of immunofluorescence

Portions of liver (1 cm × 1 cm) were fixed in 4% (v/v) neutral buffered formalin for 24 h, then dehydrated, embedded in paraffin, sectioned, stained with hematoxylin and eosin (H&E), and antibody against Nrf2 for microscopical observation and immunofluorescence.

### Protein extraction and Western blotting

Total protein of animal tissue and L02 cells was extracted from liver tissue using protein extraction kits. Proteins were separated by sodium dodecyl sulfate-polyacrylamide gel electrophoresis for approximately 2 h at 40 mA, transferred onto a polyvinylidene fluoride membrane, blocked with 5% (v/v) skim milk for 1 h at room temperature, incubated separately with primary antibodies against P62, Beclin1, LC3B, Nrf2, p-Akt, and p-FOXO3a, and primary antibodies against *β*-actin at 4°C overnight, washed with buffer, followed by incubation with the corresponding secondary antibodies, and developed using the BeyoECL Star Western blotting detection reagent.

### MTT assay

Cells were plated in 96-well plates with 5 × 10^3^ cells/well and incubated overnight for confluency. After treating with CQ (10 *μ*M), PU (50 *μ*M), CCl_4_ (30 *μ*M), and 0.1% DMSO as described above, MTT reagent (5 mg/mL) was added and incubated at 37°C in the dark for 4 h. Then, the medium was removed, and formazan crystals were dissolved in DMSO (100 *μ*M/well). The absorbance of the dissolved crystals was measured at 490 nm using a microplate reader.

### Statistical analysis

Statistical analyses were conducted using SPSS (ver. 18.0; SPSS, Inc., Chicago, IL, USA) and GraphPad Prism software (ver. 7.0; Graph Pad Software, Inc., San Diego, CA, USA). Continuous data are expressed as means ± standard deviation (SD). Normality was evaluated using the Shapiro-Wilk test. Groups were compared through one-way ANOVA followed by a *post hoc* test, the least significant difference *t*-test, with *P* < .05 and *P* < .01 taken to reflect significance.

## Results

### Establishment of liver injury model induced by CCl_4_

**FIG. 1.**
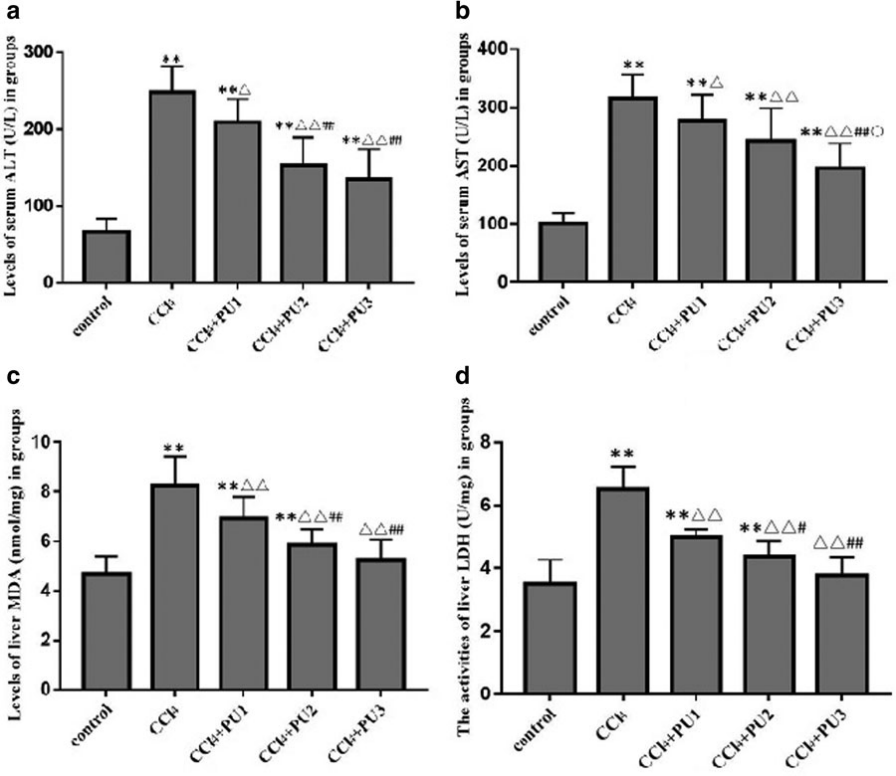
CCl_4_-induced liver injury in mice. **(a)** Levels of serum ALT in groups. **(b)** Levels of serum AST in groups. **(c)** The levels of liver MDA in groups. **(d)** The activities of liver LDH in groups. **P < .01, compared with control group; ^▵▵^*P* < .01, ^▵^*P* < .05, compared with CCl_4_ group; ^##^*P* < .01, ^#^*P* < .05, compared with CCl_4_+PU1 group; ^○^*P* < .05, compared with CCl_4_+PU2 group. ALT, alanine aminotransferase; AST, serum aspartate aminotransferase; CCl_4_, carbon tetrachloride; LDH, lactate dehydrogenase; MDA, malondialdehyde; PU, punicalagin.

**FIG. 2.**
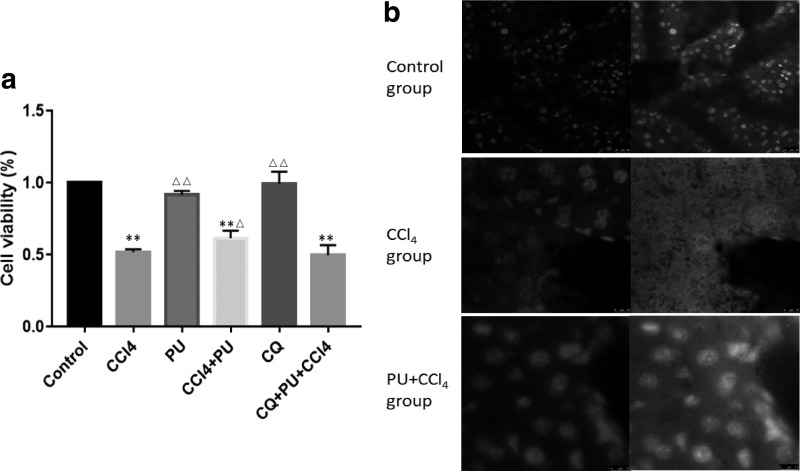
Cell viability in L02 cells and immunofluorescence detection in mice liver. **(a)** Cell viability; **(b)** immunofluorescence of Nrf2. **P < .01, compared with control group; ^▵▵^*P* < .01, ^▵^*P* < .05, compared with CCl_4_ group. NrF2, nuclear factor E2-related factor.

To explore the potential role and the mechanism of PU in CCl_4_-induced hepatic injury, the CCl_4_-induced liver injury model was established using ICR mice and L02 cells. As shown in Table 1, compared with the control group, the liver index of the CCl_4_ group was significantly higher (*P* < .01). Biochemical test was also performed to examine the serum level of ALT and AST, which are crucial indicators of liver function. As expected, the CCl_4_ group had higher levels of serum ALT and AST than the control group (Fig. 1a, b) (*P* < .01). Liver MDA level and liver LDH activity were detected using kits. We can observe from Figure 1c and d that liver MDA level and LDH activity were significantly increased in ICR mice challenged with CCl_4_ (*P* < .01). MTT assay also showed that the survival of L02 cells treated with 30 μM CCl_4_ decreased compared with control group (*P* < .01) (Fig. 2a).

**Table 1.** Body Weight and Liver Index of the Mice in Various Groups

Group	Body weight (g)		Liver index (g/100 g)
	Before gavage	After gavage	
Control group	24.87 ± 0.94	37.94 ± 4.23	4.45 ± 0.25
CCl_4_ group	24.68 ± 1.15	35.52 ± 3.21	5.29 ± 0.27^**^
CCl_4_+PU1	24.77 ± 1.26	37.06 ± 2.87	4.69 ± 0.41^▵▵^
CCl_4_+PU2	24.90 ± 1.24	36.28 ± 3.52	4.15 ± 0.37^▵▵##^
CCl_4_+PU3	24.78 ± 1.18	37.17 ± 4.20	4.41 ± 0.36^▵▵^

The data represent the mean ± SD of 10 mice per group.

^**^ *P* < .01, compared with control group.

^▵▵^ *P* < .01, compared with CCl_4_ group.

^##^ *P* < .01, compared with CCl_4_+PU1 group.

CCl_4_, carbon tetrachloride.

**FIG. 3.**
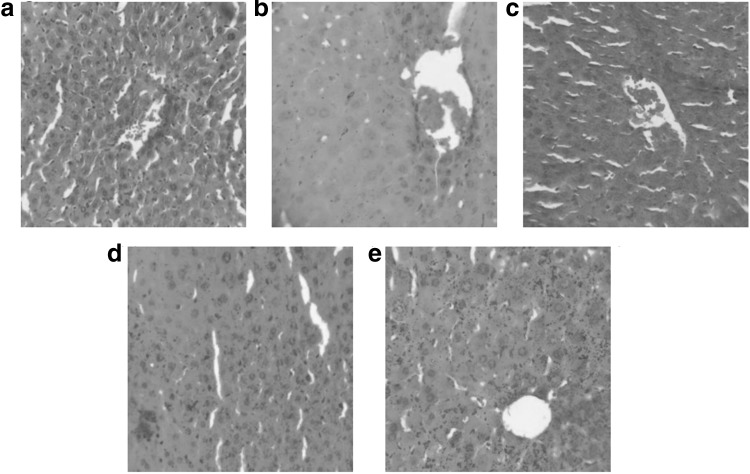
Protective potential effect of PU on liver histopathology of mice in various groups (40 × magnification with H&E stain). **(a)** Control group; **(b)** CCl_4_ group; **(c)** CCl_4_+PU1 group; **(d)** CCl_4_+PU2 group; **(e)** CCl_4_+PU3 group. H&E, hematoxylin and eosin.

Liver tissues were stained with H&E, examined microscopically, and photographed (40 × magnification) to provide further evidence supporting our biochemical assessment. As shown in Figure 3a, the physiological form of healthy mouse liver was normal; the hepatocytes, hepatic artery and vein, hepatic bile duct were neatly and orderly arranged. Exposed to CCl_4_, the liver cells were disorganized and exhibited necrosis and inflammatory infiltration indicative of hepatotoxicity (Fig. 3b). These results reflected the severe impaired liver function in the CCl_4_ group, which revealed that the model we established was effective and appropriate for further study.

### PU improved the hepatic injury induced by CCl_4_

Cell viability showed no significant difference between control group and cells only treated with PU (*P* > .05), which indicated low toxicity. Furthermore, viability of cells treated with PU+CCl_4_ was significantly higher than that of CCl_4_ group (*P* < .01) (Fig. 2). To examine the effect of PU against the hepatotoxicity of CCl_4_, the above-mentioned liver index, serum ALT and AST levels, liver LDH activity and cell viability were also detected (Figs. 1 and 2).

The body weight among the various groups did not change significantly before and after the 4-week treatment with PU by gavage (*P* > .05). Treatment with PU resulted in significantly lower liver index in CCl_4_+PU1 (20 mg/kg), CCl_4_+PU2 (40 mg/kg), and CCl_4_+PU3 (80 mg/kg) groups (*P* < .01). The mice in the CCl_4_+PU2 group had the lowest liver index (*P* < .01) of all the CCl_4_ treated groups, and there was no significant difference in the liver index between the control group and the PU group (*P* > .05). Compared with the CCl_4_ group, the serum ALT and AST levels, and liver LDH activities of groups treated with different doses of PU decreased gradually (Fig. 1), suggesting a protective effect of PU on liver function. Treatment with PU at various concentrations prevented the pathological changes and inflammation of hepatocytes (Fig. 3). These results indicated that PU protected against the hepatic injury induced by CCl_4_ in L02 cells and liver of ICR mice.

### PU enhanced the antioxidant activities in the liver injury model induced by CCl_4_

**FIG. 4.**
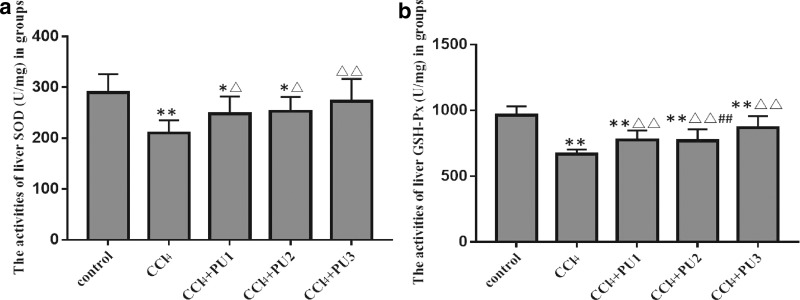
PU exerted antioxidant activities against CCl_4_-induced liver injury in mice. **(a)** The activities of liver SOD in groups; **(b)** the activities of liver GSH-Px in groups. The data represent the mean ± SD of 10 mice per group. **P < .01, *P < .05, compared with control group; ^▵▵^*P* < .01, ^▵^*P* < .05, compared with CCl_4_ group; ^##^*P* < .01, compared with CCl_4_+PU1 group. GSH-Px, glutathione peroxidase; SOD, superoxide dismutase.

Oxidative stress plays an important role in the progression of liver damage. Our results also verified that liver SOD and GSH activities were lower in CCl_4_ group, and liver MDA levels increased compared with the control group (*P* < .01) (Fig. 4).

Compared with the CCl_4_ group, the mice that received PU (20, 40, and 80 mg/kg i.g.) had higher activities of SOD and GSH, but simultaneously lower levels of liver MDA (*P* < .01).

**FIG. 5.**
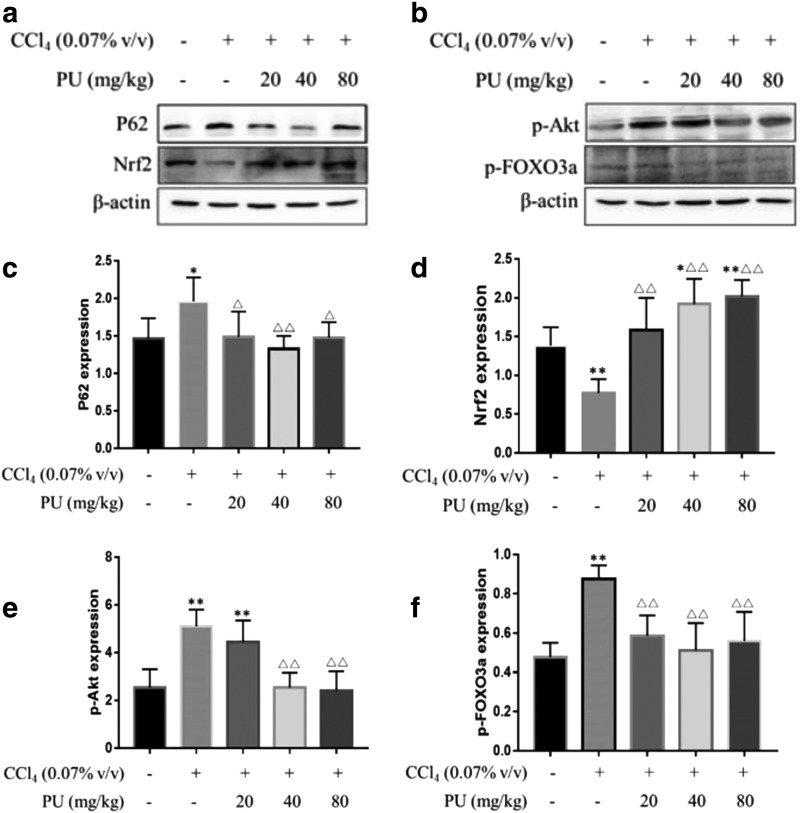
The effect of PU on the P62 Nrf2 p-Akt and p-FOXO3a proteins expression in liver homogenates in mice. **(a)** Pictures of the P62 and Nrf2 protein expression in groups. **(b)** Pictures of the p-Akt and p-FOXO3a proteins expression in groups. **(c)** The expression of liver P62 protein in groups. **(d)** The expression of liver Nrf2 protein in groups. **(e)** The expression of liver p-Akt protein in groups. **(f)** The expression of liver p-FOXO3a protein in groups. The data represent the mean ± SD of 10 mice per group. **P < .01, *P < .05, compared with control group; ^▵▵^*P* < .01, ^▵^*P* < .05, compared with CCl_4_ group. p-FOXO3a, phosphorylation of forkhead box O 3a.

Furthermore, detection of Nrf2 in animal liver, the main factor regulating the expression of antioxidant protein, was completed by western blot (Fig. 5d). In the CCl_4_ group, expression of Nrf2 decreased markedly than in control, meaning that CCl_4_-induced oxidative stress was mediated by Nrf2. However, in animals fed with PU, the level of Nrf2 rose to near-normal levels. The above findings revealed that PU suppressed Nrf2-mediated oxidative stress in liver injury induced by CCl_4_. Besides, as shown in Figure 2b, the expression of Nrf2 was measured through immunofluorescence stained with DAPI (nuclei) and antibody of Nrf2, which showed more expression of Nrf2 in cell nucleus with pretreatment of PU than CCl_4_ group.

### PU upregulated autophagy in the liver injury model induced by CCl_4_

**FIG. 6.**
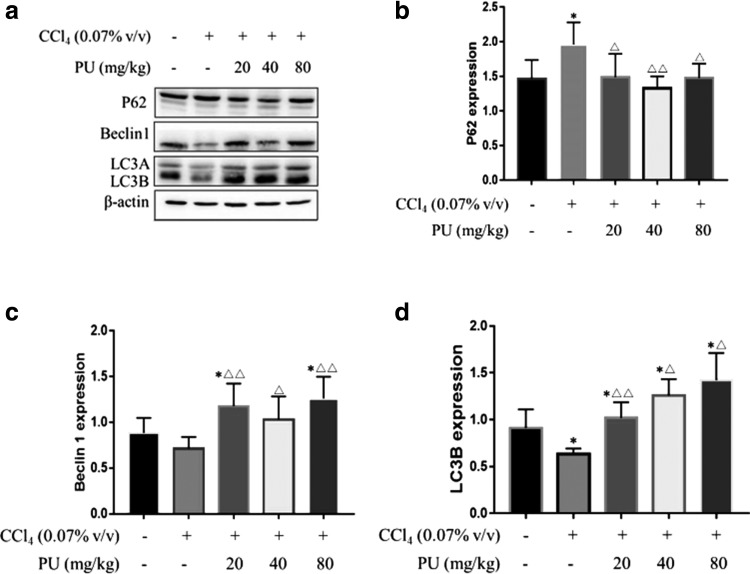
The effect of PU on the P62, Beclin1, and LC3B proteins expression in liver homogenates in mice. **(a)** Pictures of the P62, Beclin1, and LC3B protein expression in groups. **(b)** The expression of liver P62 protein in groups. **(c)** The expression of liver Beclin1 protein in groups. **(d)** The expression of liver LC3B protein in groups. The data represent the mean ± SD of 10 mice per group. *P < .05, compared with control group; ^▵▵^*P* < .01, ^▵^*P* < .05, compared with CCl_4_ group.

To further explore the mechanism of PU against CCl_4_-induced oxidative stress in liver, situations of autophagy flux and autophagy were evaluated. As shown in Figure 6c and d, Beclin1 and LC3B expressions were low whereas soluble P62 was high in the CCl_4_ group, implying that CCl_4_ suppressed the first stage of autophagy, macroautophagy, and autophagy flux as well. It can be speculated that CCl_4_ was more likely to block the induction of autophagy. After treatment with PU, autophagy flux and autophagy of hepatocytes were promoted, according to the higher level of Beclin1, LC3B and lower expression of soluble P62 in the CCl_4_+PU group than in the CCl_4_ group (Fig. 6c, d).

In addition to the increase in PU dosage, LC3B was multiplied gradually. In the CCl_4_+PU2 group, the levels of soluble P62 and Beclin1 were lower compared with those in the other two experimental groups. This phenomenon identified strong effect of PU on macroautophagy rather than the activation of autophagy. Moreover, inappropriate concentrations of PU were preferred for the activation of autophagy and low autophagy flux activity. This suggested that PU might exert antioxidative effect primarily through upregulation of macroautophagy and autophagy flux.

**FIG. 7.**
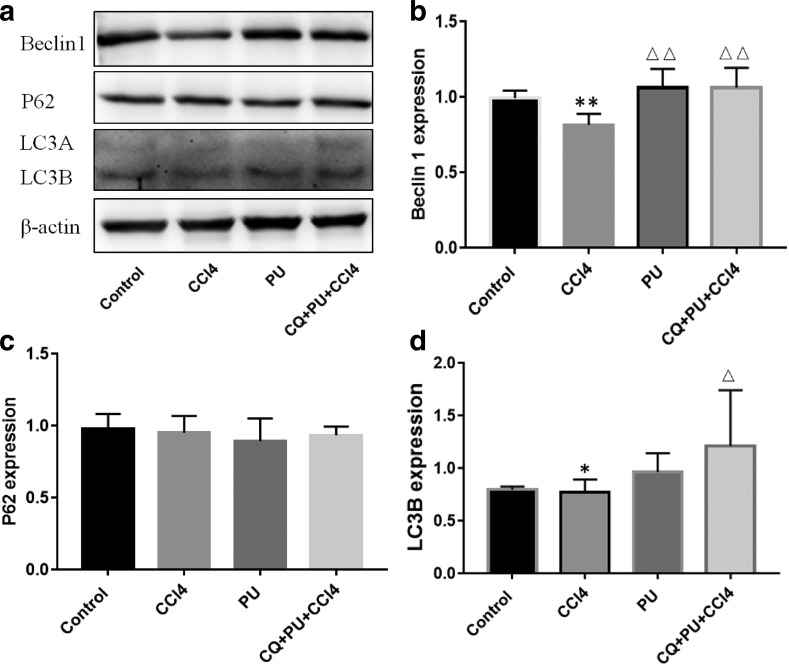
The effect of PU on the Beclin1, P62 and LC3B proteins expression in L02 cells. **(a)** Pictures of Beclin1, P62 and LC3B proteins expression in groups. **(b)** The expression of liver Beclin1 protein expression in groups. **(c)** The expression of liver P62 protein expression in groups. **(d)** The expression of liver LC3B protein expression in groups. The data represent the mean ± SD of 10 mice per group. **P < .01, *P < .05, compared with control group; ^▵▵^*P* < .01, ^▵^*P* < .05, compared with CCl_4_ group.

It also showed the effect of PU on inducing autophagy in L02 cells (Fig. 7). Although there was no significant difference in p62 and LC3B expression between control and CCl_4_ groups, Beclin1 expression was lower in the CCl_4_ group. Compared with the CCl_4_ group, Beclin1 expression was significantly higher in the group pretreated with PU, especially in the group treated with CQ. CQ was shown to be an inhibitor of autophagy through inhibiting autophagic degradation in the lysosomes, which might account for the higher expression of LC3B in cells.

### PU regulated autophagy by p-Akt and p-FOXO3a

To tentatively investigate how PU regulates autophagy in hepatocyte against CCl_4_-induced injury, we examined the expression of p-Akt and p-FOXO3a (Fig. 5e, f). Compared with the control group, the CCl_4_ group and low-dose PU group had significantly higher expression of p-Akt (*P* < .05), and the CCl_4_ group had significantly higher expression of p-FOXO3a (*P* < .05). Compared with the CCl_4_ group, the expressions of p-Akt were significantly lower in the PU2 and PU3 groups (*P* < .05). Meanwhile, the expression of p-FOXO3a was significantly lower in PU group than in the CCl_4_ group (*P* < .05). These results indicated that PU might upregulate autophagy in hepatocyte through suppressing the expression of p-Akt and p-FOXO3a.

## Discussion

CCl_4_, a colorless, volatile nonflammable liquid, is a haloalkane widely used in industry and chemical laboratories to produce commercial fats, lacquers, and other organic compounds. It is toxic to both humans and animals. Since the metabolism of CCl_4_ occurs mainly in liver cells, and the alterations of symptoms, medical indices, and pathological morphology of liver induced by CCl_4_ are similar to those induced by some drugs (*e.g*., acetaminophen),^13^ CCl_4_ is often used as one of the popular liver injury models to explore possible pathological mechanisms contributing to the amelioration of liver diseases.

CYP450, especially CYP2E1, can decompose CCl_4_ to the radical ^•^CCl_3_, which not only binds to cellular molecules, triggering accumulation of triglycerides, but also reacts with oxygen to form CCl_3_OO^•^, a reactive oxygen species (ROS). ROS can result in lipid peroxidation that affects the permeabilities of the mitochondrial, endoplasmic reticulum and plasma membranes, in turn affecting the cellular calcium balance and contributing to subsequent cell damage.^14^

MDA is an end-product of liver lipid peroxidation. High MDA concentration in liver is an indicator of lipid peroxidation induced by CCl_4_. To alleviate oxidative stress, the antioxidant enzyme SOD converts the highly reactive superoxide radical to H_2_O_2_, and subsequently GSH-Px decomposes H_2_O_2_ into H_2_O to reduce the damage caused by reactive hydroxyl radicals. Moreover, increasing liver LDH level, an enzyme of the glycolytic pathway, is another indicator of hepatocyte damage and loss of functional integrity.

ALT and AST are located principally in hepatocyte cytoplasm, and are released into the circulation after cell damage.^15^ Thus, CCl_4_ clearly caused liver injury, as also shown by the elevated liver index and the histopathological changes in this study. We found that PU could attenuate CCl_4_-induced injury, which was evidenced by reducing the CCl_4_-induced increases of the serum ALT and AST levels, liver MDA level, liver LDH activities, the decreases in liver SOD and GSH-Px activities, and the amelioration of hepatic histopathology. The SOD, LDH activities, and MDA levels returned to near-normal levels in the CCl_4_+PU3 group. Therefore, PU exerted an antioxidative effect to protect liver cells from CCl_4_-induced oxidative stress.

Nrf2 plays crucial roles in redox and antioxidation regulation. Nrf2 usually forms an inactive complex with Keap1, a molecular sensor of electrophiles and pro-oxidants in the cytoplasm.^16^ Under stress, the affinity between Nrf2 and Keap1 is reduced. After separation from Keap1, Nrf2 is translocated to the nucleus to form a heterodimer with a small Maf protein that binds to antioxidant response elements and electrophile response elements. Then, Nrf2 can induce the encoding of genes for many antioxidant and detoxifying enzymes.^17^ Nrf2 has been studied in the context of disease prevention and treatment. PU protected HepG2 cells through Nrf2 activation.^18^ In this study, Nrf2 expression was increased in the PU groups compared with the other groups. It further proved that PU has high antioxidative activities, associated with the enhanced activities of enzymes such as SOD and GSH-Px. Hence, other pathways and targets should be examined in the context of Nrf2 upregulation induced by PU.

P62/SQSTM1 (P62), a 62-kDa protein, is a multifunctional signaling hub and an autophagy adaptor that takes part in several signaling pathways involving not only Nrf2 but also mammalian target of rapamycin (mTOR), mitogen-activated protein kinase, and NF-*κ*B.^19^ Katsuragi *et al.* found that P62, a Nrf2 target, could activate Nrf2 by binding to Keap1. P62 has many domains, one of which, the Keap1-interacting region (KIR), is associated with the Keap1/Nrf2 pathway. Phosphorylation of serine 62 of KIR by P62 induced by various stresses notably increased binding of P62 and Keap1, reducing binding between Keap1 and Nrf2 and increasing Nrf2 nuclear translocation to induce expression of cytoprotective Nrf2 targets.^20^ This study showed that the P62 expression significantly increased in the CCl_4_ group compared with the control group, and decreased in the PU groups compared with the CCl_4_ group. Considering the changes in Nrf2 expression, the changes in P62 expression might be caused by the binding of P62 to Keap1.

Moreover, P62 binds to microtubule-associated protein 1A/1B-light chain 3 (LC3) proteins through the LC3-interacting region (LIR), a short linear sequence, resulting in selective autophagic clearance of ubiquitinated protein aggregates and organelles.^19^ LC3 is a prominent marker of late macroautophagy, which is one of the main forms of autophagy.^21^ Autophagy is a self-digestive process during which damaged organelles or useless proteins are removed with systematic recycling of unnecessary or malfunctioning cellular components. Macroautophagy is also termed selective autophagy, characterized by the formation of autophagosomes, double-membrane structures, separating damaged organelles or useless proteins from the cytoplasm.

Autophagy includes four stages: induction, nucleation, extension, and maturation.^22^ Beclin1 plays an important role in the first stage. It binds to phosphatidylinositol 3-kinase class III (PtdIns3KC3) and Vps15, forming PtdIns3KC3 complex, to induce autophagosome formation.^23^ After that, a cup-shaped double membrane, called isolation membrane, emerges in the cytoplasm. The Atg12-Atg5-Atg16 complex locates on the isolation membrane, and then LC3 conjugated to phosphatidylethanolamine, also called LC3B, locates on the membrane. LC3B may facilitate the closure of the isolation membrane; therefore, it is regarded as an index for the late stage of autophagy.^21^ P62, an autophagy adaptor promoting the degradation of autophagic cargos, also contains other domains, such as phox and bem1 (PB1), and a ubiquitin-associated (UBA) domain. The PB1 domain participates in the formation of autophagosome. P62 interacts with LC3 through the LIR domain to facilitate the clearance of autophagic and ubiquitinated cargos,^19^ and intracellular P62 protein can be degraded through autophagy and the ubiquitin proteasome system related to the UBA domain.

It has been demonstrated that autophagy may be helpful to protect liver from ethanol-induced hepatotoxicity,^24^ nonalcoholic fatty liver disease,^25^ and CCl_4_-induced liver fibrosis.^26^ According to the animal experiment, we found that the PU groups had significantly higher expressions of Beclin1 than the CCl_4_ group. Moreover, the CCl_4_ group had significantly lower expression levels of LC3B than the control group, whereas the PU groups had significantly higher expression level of LC3B than the control group and CCl_4_ group. In consequence, PU may induce autophagy against CCl_4_-induced liver injury.

*In vitro*, we also observed higher expressions of Beclin1 and LC3B in the groups pretreated with PU than in the CCl_4_ group. When pretreated with CQ, the inhibitor of autophagic degradation, the expression of LC3B further increased. As the results show, autophagy plays an important role in alleviating the hepatic injury induced by CCl_4_. More work is needed to further elucidate how autophagy is regulated by PU.

Akt, also known as Akt kinase, is involved in the regulation of various signaling pathways for cell metabolism, cell proliferation, growth, survival, and apoptosis.^27^ It is reported that the Akt pathway can inhibit autophagy through activation of mTOR and phosphorylation of Beclin1.^28^ p-Akt at S473 and T308 is regarded as the active form of Akt to activate its downstream pathways.^29^ This study showed that PU might induce autophagy through the regulation of the Akt pathway.

Furthermore, it is reported that FOXO3a, one of the FOXO transcription factors, can be regulated by the Akt pathway to promote cell proliferation, apoptosis, stress-resistant effect, autophagy, etc. For example, it can increase the expression of MnSOD and catalase against oxidative stress. The p-FOXO3a by Akt leads to its inactivation through relocating at cytoplasm from nucleus.^30^ We found that CCl_4_ might activate the Akt pathway and promote the phosphorylation of FOXO3a, while PU might suppress the Akt pathway and the expression of p-FOXO3a.

In conclusion, the results of this study showed that PU prevented phosphorylation of AKT/FOXO3a, which facilitated Beclin1 expression, thereby activating autophagy. Together, LC3B and insoluble P62 accumulation caused macroautophagy and autophagy flux to be amplified vastly. After that, Nrf2 was upregulated and antioxidant activity enhanced, resulting in reversal of CCl_4_-induced hepatic injury (Fig. 5). However, more work is needed to further elucidate how autophagy is controlled in this process. The findings provide new perspectives for the exploration of PU against liver injury.
